# COVID-19 myth-busting: an experimental study

**DOI:** 10.1186/s12889-021-12464-3

**Published:** 2022-01-19

**Authors:** Aimée Challenger, Petroc Sumner, Lewis Bott

**Affiliations:** School of Psychology, Cardiff University, Cardiff, UK

**Keywords:** Misinformation, COVID-19, Myth busting, Myth correction, Infodemic

## Abstract

**Background:**

COVID-19 misinformation is a danger to public health. A range of formats are used by health campaigns to correct beliefs but data on their effectiveness is limited. We aimed to identify A) whether three commonly used myth-busting formats are effective for correcting COVID-19 myths, immediately and after a delay, and B) which is the most effective.

**Methods:**

We tested whether three common correction formats could reduce beliefs in COVID-19 myths: (i) question-answer, ii) fact-only, (ii) fact-myth. *n* = 2215 participants (*n* = 1291 after attrition), UK representative of age and gender, were randomly assigned to one of the three formats. *n* = 11 myths were acquired from fact-checker websites and piloted to ensure believability. Participants rated myth belief at baseline, were shown correction images (the intervention), and then rated myth beliefs immediately post-intervention and after a delay of at least 6 days. A partial replication, *n* = 2084 UK representative, was also completed with immediate myth rating only. Analysis used mixed models with participants and myths as random effects.

**Results:**

Myth agreement ratings were significantly lower than baseline for all correction formats, both immediately and after the delay; all β’s > 0.30, *p*’s < .001. Thus, all formats were effective at lowering beliefs in COVID-19 misinformation.

Correction formats only differed where baseline myth agreement was high, with question-answer and fact-myth more effective than fact-only immediately; β = 0.040, *p* = .022 (replication set: β = 0.053, *p* = .0075) and β = − 0.051, *p* = .0059 (replication set: β = − 0.061, *p* < .001), respectively. After the delay however, question-answer was more effective than fact-myth, β = 0.040, *p* =. 031.

**Conclusion:**

Our results imply that COVID-19 myths can be effectively corrected using materials and formats typical of health campaigns. Campaign designers can use our results to choose between correction formats. When myth belief was high, question-answer format was more effective than a fact-only format immediately post-intervention, and after delay, more effective than fact-myth format.

**Supplementary Information:**

The online version contains supplementary material available at 10.1186/s12889-021-12464-3.

## Background

The COVID-19 pandemic has spawned an abundance of misinformation [1–4], described by the World Health Organization (WHO) as an ‘infodemic’ [5]_._ In many countries, misinformation preceded the outbreak of COVID-19 infections and posed a serious threat to public health [6]. False statements such as “prolonged use of face masks cause health problems” [7], “over 90% of positive COVID-19 tests are false” [7] and “the new COVID-19 vaccine will alter your DNA” [7] reduce compliance with health advice [8] and oblige health teams to compete with science denialism groups. For this reason, the WHO identifies COVID-related misinformation 24 h a day [9] and provides ‘myth-busting’, as do many WHO member countries, such as the UK [10] and Brazil [11], and prominent online platforms (e.g., The Guardian [12], BBC [13], and fact checker websites [7, 14, 15]).

But correcting misinformation is difficult. Misinformation can sway reasoning long after attempts have been made to correct it [16–23]. Health campaigns must therefore optimise their materials to maximise belief change. This requires successfully linking the correction with misinformation in the mind of the reader [24]. Traditionally, myth-busting campaigns have done this explicitly by naming the myth as well as providing a rebuttal (“Myth: the COVID-19 vaccine is mandatory. Fact: the COVID-19 vaccine is not mandatory…”). This approach is used extensively in public health (e.g., influenza [25], smoking [26] and has been applied to COVID-19 myths [7, 13, 14, 27–30].

However, there have been fears that repeating the myth makes the misinformation more familiar and therefore more likely to be considered true [18]. This phenomenon could lower campaign effectiveness [18], and more recent campaigns, such as those by the WHO, have used approaches that either avoid repeating the myth entirely (*fact-only*, “The new COVID-19 vaccine will not alter your DNA”) or implicitly link the myth with the correction using a question-answer format (*question-answer*, “Does the new COVID-19 vaccine alter your DNA? No…”). In contrast to these approaches, recent studies question the need to omit the myth [31–33], although current guidance recommends placing the myth after, rather than before, the rebuttal [34]. Indeed, including myths can sometimes have positive effects on belief change [29, 35].

In this study we compared three approaches to myth-busting to establish whether health campaigns might be most effective when they include the myth, omit the myth, or use a question-answer format. We used a randomised trial with a representative sample.

### Facts and myths vs only facts

A central question in myth-busting is whether to repeat myths in the myth-busting materials or to present only correcting facts. Early studies suggested that repeating myths had a detrimental effect [18]. It was argued that they risked making the myths more familiar [36], and that they promoted shallow processing of the material [37]. For example, Skurnik, Yoon & Schwarz [38] found that after a 30-min delay, participants in a flu myth-busting condition mistakenly mislabelled myths as facts. They also found that intention to obtain the influenza vaccine was lowered following corrective information that included statements of the myth (a ‘backfire’ effect). Such backfire effects led to advice not to make explicit reference to myths, but present only facts [18, 39].

But recent work presents a more muted conclusion. Familiarity effects have proven elusive [31–33] and difficult to replicate [40]. For example, Swire et al. [31] presented participants with a series of true and false claims (myths) that were subsequently affirmed or corrected. They measured the corresponding change in belief and found no evidence of backfire effects at short or long delays, or in older people (whose ability to recall information using strategic memory processes is typically less efficient than younger people’s).

Recent studies have also found advantages for restating the myth during correction, both immediately and after a delay [31, 33, 36, 41]. A limitation of these studies, however, is that they were not designed with Public Health interventions in mind. Instead, they focused on fake news headlines or stories, or general claims that were then fact-checked, e.g., “The national animal of Scotland is the unicorn” (true). For example, the study generally cited to support inclusion of the myth is Ecker, Hogan & Lewandowsky [42], who used a continued influence paradigm modelled on misinformation retraction in news media. Participants read novel news stories (e.g., about a wildfire) that included crucial information (how the fire started) that was later retracted. Retraction that explicitly stated the original information ("It was originally reported that the fire had been deliberately lit, but authorities have now ruled out this possibility. After a full investigation and review of witness reports, authorities have concluded that the fire was set off by lightning strikes") was more effective than retraction that did not ("After a full investigation and review of witness reports, it has been concluded that the fire was set off by lightning strikes"). The materials in health campaigns and social media generally contain much less information than the whole news stories used in continued influence paradigms, are aimed at familiar myths rather than novel news, correct myths after a much longer delay, and have a more diverse audience than Ecker et al.’s participants (*n* = 60 per condition, Psychology students).

In summary, the prevailing view is that including myths as well as facts is more effective at changing beliefs than including only facts. Nonetheless, the variability in findings, and differences between health campaigns and experimental investigations motivated our dedicated COVID-19 study designed with the specific purpose of providing advice for health campaigns.

### Question-answer

Explicitly including the myth in a correction provides a cue that there is a conflict between the facts and pre-existing beliefs. An alternative approach to myth-busting is to use a question format that implicitly cues the myth (“Will the new COVID-19 vaccine alter your DNA? No…”). This format prompts the reader to internally retrieve the answer to the myth question. Conflict is potentially generated between the retrieved and the provided answer and resolved by belief revision.

It is unknown whether implicitly cueing the myth, as in question-answer, produces greater correction of the myth than explicitly doing so. Greater correction might arise because interrogatives yield more engagement or intrinsic motivation than declarative statements [43]. For example, “Will I ….?” motivates more goal directed behaviour than “I will ….”, and rhetorical questions are more effective at encouraging elaborative processing of material than declaratives [37, 44]. On the other hand, implicitly cueing the myth risks the reader failing to access relevant representations. For example, the reader may not expend sufficient processing time to retrieve the correct answer to the question [45]. If this happens, there would be no coactivation of the myth and the correction, and so belief revision would not arise [24].

The question-answer format is currently deployed by the WHO, amongst others, to combat coronavirus misinformation [30]. One study has used this approach, using a WHO infographic to correct the myth that garlic is a cure for coronavirus [46], with mixed results: there was no significant overall effect on misinformation belief, and there was a backfire effect for older adults (55+) (although it was not the authors’ purpose to compare question-answer format to any other approach; they were comparing age groups in UK and Brazil).

### Study rationale and outline

In sum, question-answer, fact-only and fact-myth formats are all currently deployed in an attempt to correct COVID-19 misinformation. There are reasons to favour each. Fact-myth presents an explicit link between pre-existing beliefs and corrective material and so may facilitate the detection of conflict, but risks making the myth familiar. Fact-only avoids making the myth more familiar, but risks failing to link pre-existing beliefs and corrective material. Question-answer invites an implicit link between myth and correction that may be more engaging and could yield better recall, but for the same reason it could boost myth familiarity.

To identify which format is most effective at generating belief change in COVID-19, we compared their effectiveness using a randomised trial with a representative UK sample. Participants read myths/facts and appropriate corrections and then answered inference questions testing their agreement with the myths.

**Fig. 1:**
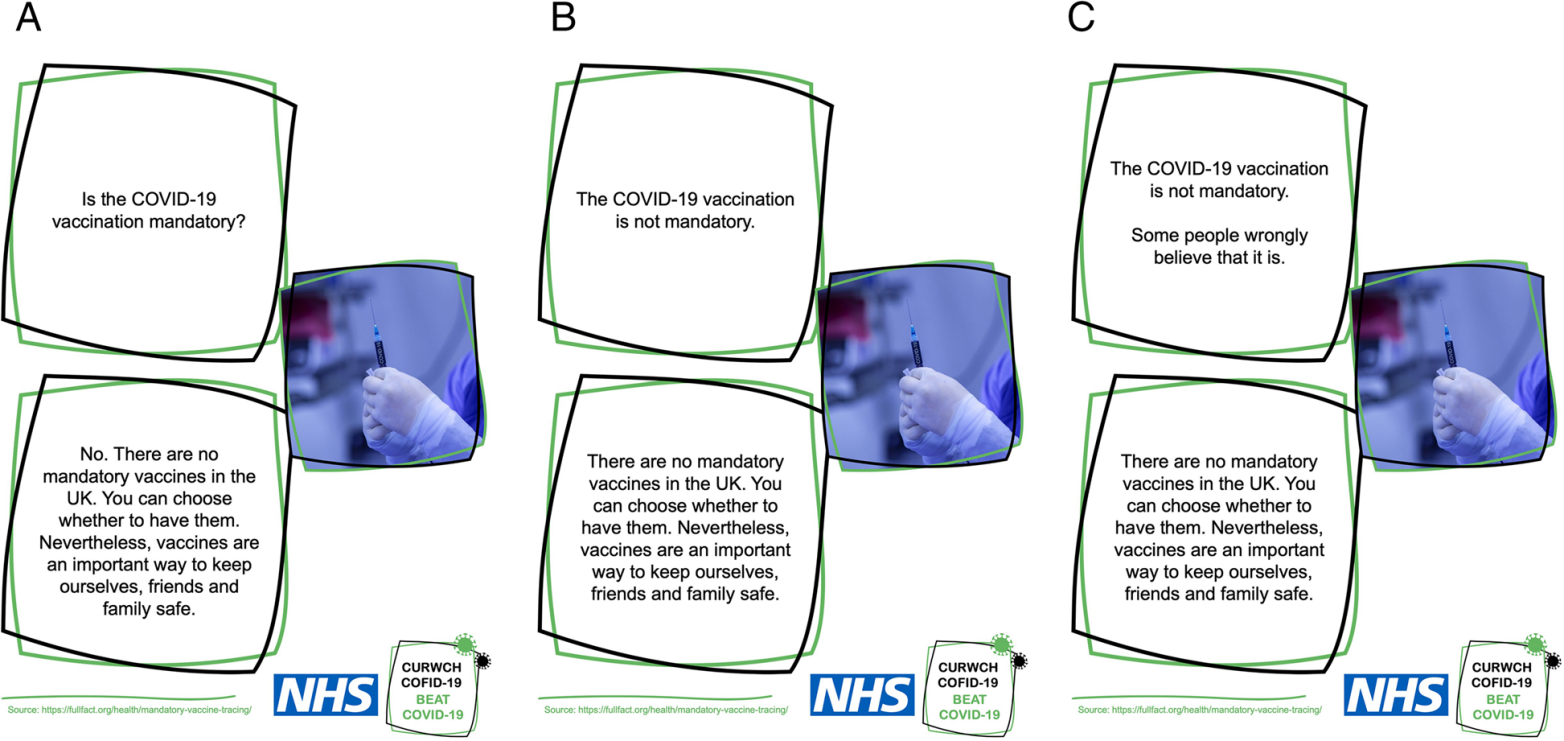
Example correction graphics. There were three correction format conditions: **A** Question-answer **B** Fact-only **C** Fact-myth. Each graphic had two boxes. The first contained the intervention material, the second the supporting explanation statement (and the answer, i.e., yes/no, in the case of question-answer)

There were three between-subject conditions (i) *Question-answer,* (ii) *Fact-only,* (iii) *Fact-myth* (Fig. 1). The materials were designed to be useable and relevant to public health but also follow the most recent advice [34]. When including the myth, traditional public health myth-busting typically present a myth first and then a correction (myth-fact), but the current advice [34] is to place the fact first and then the myth (fact-myth). We followed this advice.

Participants were tested prior to correction (baseline) to establish baseline beliefs to act as a (repeated measures) control condition. Participants were then tested immediately after correction (timepoint 1) and after a delay of at least 6 days (timepoint 2). This enabled us to answer the following main research questions:

1. Which formats are effective immediately and after a delay? That is, does each format lower agreement with myths (effective correction), increase agreement (a backfire effect), or neither?
2. What is the most effective myth correction format?

In exploratory work, we also investigated how age interacted with our research questions.

## Methods

### Ethics information

The project was approved by Cardiff University School of Psychology’s Ethics Committee (EC.19.07.16.5653GR2A4). Participants consented at the beginning of the study and received payment and debrief after participation.

### Preregistration

The study was pre-registered at (https://osf.io/huz4q/).

### Design and materials

#### Myth selection

We ran two short surveys to select real-world COVID-19 myths as materials (Table SI.M.1). Together these surveys yielded 11 myths for the main study (see Table 1). The first survey tested a list of 39 myths sourced from the WHO’s COVID-19 myth-busters list [30] and fact checker websites [7, 15]_._ Myths were included if they had potential to influence readers’ behaviour. For example, the myth that “500 lions were released into the streets to prevent people from leaving their houses during lockdowns in Russia” [7] was not included as it was unlikely to affect behaviour in the UK. The myth that “gargling with salt water can prevent COVID-19” [7] was included as it had the potential to change behaviour. Final myths were selected following discussion and consensus of the team.

**Table 1:** Myths used in the study

	Myth
1	Seasonal colds and flu are wrongly being counted as COVID-19 cases
2	If your symptoms are mild, you can self-isolate and don’t have to take a test
3	Lockdowns don’t work
4	Thermal scanners can detect people who have COVID-19 but aren’t showing symptoms
5	The flu is more deadly than COVID-19
6	The COVID-19 vaccination is mandatory
7	Lockdowns are due to healthy people getting tested
8	Ultra-violet lamps should be used to disinfect hands
9	Face masks do not reduce the transmission of infection
10	Wearing a face mask can lead to increased levels of carbon dioxide in the blood
11	As soon as you have had the vaccine, you can go back to your normal life

Fifty participants recruited from the online participant panel Prolific [47] rated how much they agreed with each myth, alongside four COVID-19 facts, in a random order, using a pointer on a visual analogue scale from “Strongly disagree (0)” to “Strongly agree (100)”. We selected myths with above 20% average agreement to be included in this study. This process yielded five myths.

We repeated the study with a new set of 18 myths (again those with behavioural relevance) from the WHO [30] and fact checker websites [7, 15, 48, 49] and an additional 50 participants (Prolific). One participant was removed for giving the same response [50] to all questions. Again, we selected all myths with above 20% average agreement, except for one because there was subsequent scientific debate about whether it was partially true (the effects of Vitamin D). This yielded six myths.

#### Correction graphics

Graphics were designed to conform to current myth-busting advice [34]. Each graphic (Fig. 1) therefore contained source information, including an NHS and COVID-19 logo, and a supporting explanation statement that gave an alternative to the myth (Table SI.M.1). We also included a non-probative image (an image that is related to the claim but does not give extra information about the claim’s veracity), since such images are often included in Public Health Campaigns [50]. The same image was used in each format because engagement can be increased even by non-informative images [51, 52].

#### Agreement questions

Participants rated their agreement with myths in response to questions that differed in style to the correction graphics to avoid pattern matching between the two (Table SI.M.1). Agreement ratings were made on a six-point Likert scale ranging from “Strongly agree” to “Strongly disagree”. We also included 4 fact statements, to encourage participants to use the full scale (Table SI.M.2).

#### Catch questions

We used two catch questions to eliminate participants who did not read the questions. Berinksy, Margolis and Sances [53] recommend the use of multiple items to measure attention. The questions we included were “There are seven days in the week” and “The first letter of the alphabet is ‘T’”. Participants answered “True” or “False”.

#### Demographics questions

Participants were asked about age, education, ethnicity, vaccine concern, vaccine intentions and COVID-19 experiences (Table SI.M.3).

### Procedure

#### Baseline

Participants completed a short set of questions measuring demographic information and personal experiences with COVID-19. They then answered the 17 agreement questions (11 myths, 4 facts, 2 catch trials), in a random order. Participants used a six-point Likert scale.

#### Intervention

Immediately following the agreement questions, participants were randomly assigned to one of three correction formats (question-answer, fact-only or fact-myth). They then viewed the corresponding 11 correction graphics.

#### Timepoint 1

Immediately following the correction phase, participants again rated agreement with the 17 statements, in a random order.

#### Timepoint 2 (delay)

Participants completed timepoint 2 6–20 days later (*M =* 8.9 days), in which they again rated agreement in a new random order.

### Participants

We recruited participants representative for age and gender across the UK, via Qualtrics, an online participant platform. To achieve a representative sample, we applied age and gender quotas. Age 18–24: 12%, 25–34: 19%, 35–44: 18%, 45–54: 20%, 55–64: 17%, 65+: 14%. Gender male: 49%, female: 51%. Power calculations are described in the pre-registration. The main dataset consisted of 2215 participants who completed baseline and timepoint 1, of whom 1329 completed timepoint 2 (an attrition rate of 36%). Of these 38 were excluded for not meeting the minimum age requirement (18 years, *n* = 2) or for failing the catch trials (*n* = 36).

Therefore, the *n* for main analysis was 1291. Of these, 440 participants were randomly assigned to the question-answer condition, 435 to fact-only and 416 to fact-myth. 47% identified as “man”, 52% identified as “woman”. Age ranged from 18 to 89 years; 5% were 18–24 years, 16% were 25–34 years, 18% were 35–44 years, 24% were 45–54 years, 19% were 55–64 years, and 18% were aged above 65 years. 6% identified as Asian, 1.5% as Black, 89.6% as White and 2.9% as Mixed/multiple ethnic groups.

#### Replication data for timepoint 1

We also collected a partial dataset where timepoint 2 was not collected (due to an error). This data was collected 3 weeks prior to the main dataset (January 2021, the main dataset was collected in February 2021), and we use it to test for replication of the main results for timepoint 1. Two thousand two hundred seventy-five participants were recruited and 191 were excluded for not meeting the inclusion criteria described above. Six hundred ninety-one participants were randomly assigned to the question-answer condition, 687 to fact-only and 704 to fact-myth. 48% identified as “man”, 51% identified as “woman”. Participants ranged in age from 18 to 91 years; 14% 18–24 years, 21% 25–34 years, 19% 35–44 years, 19% 45–54 years, 15% 55–64 years, and 13% above 65 years. 7.7% identified as Asian, 2.2% as Black, 0.3% as Middle Eastern, 86% as White and 2.8% as mixed/multiple ethnic groups. 24% reported they were in a COVID-19 risk group, 6.6% had had a positive COVID-19 test; 8.5% reported they were healthcare workers.

### Analysis approach

Linear mixed effect (LME) models were used to analyse the data. Analysis was conducted in R using lme4 [54], lmerTest [55] and lmer_alt() (afex package [56]). Random effects for participants and myths were included in the models, allowing us to generalise across both. Effects are reported as treatment contrasts with reference level according to the reported comparison (e.g., reported effect of question-answer vs fact-myth assumes question-answer as the reference level). *p*-values were obtained via the Satterthwaite approximation.

We obtained model convergence by starting with a model that had a maximal random effects structure design (as per advice of Barr, Levy, Scheepers, & Tilley [57]), and if that did not converge, removing correlations between intercepts and slopes for myths (see [58, 59]). Model 1 (see below) converged with the maximal random effects structure but Models 2 and 3, which had many more parameters, required suppression of correlations between intercept and slopes. This led to successful model convergence in all cases. Thus, all models included slopes and intercepts for all factors where the design allowed, but not necessarily the correlations between intercepts and slopes.

Even with convergence there remained singularity warnings. We therefore tried simplifying the models by removing further random effects structure. However, this led to models that either failed to converge or were over-simplified (i.e., ignored obvious structure in the data) and consequently risked being anti-conservative (e.g., [57]). Moreover, wherever we obtained a simplified model that both converged and was absent of singularity warnings, significant effects present in the more complex models were also present in the simpler models. We therefore report the results of the most complex models that converged, as described by the models below.

#### Research question 1

To test whether each correction format lowered agreement scores at each timepoint, we used:

$$Model\;1:Myth\_agreement\sim\;timepoint\;+\;(1+timepoint\vert participant)\;+\;(1+timepoint\vert myth)$$

Where Myth_agreement is the outcome variable, and timepoint is a fixed factor (baseline, timepoint 1, timepoint 2). Random effects (identified to the right of the pipe symbol, |) include intercepts (identified by 1 left of |) and slopes (identified by named factors after 1+), and correlations between the two. Model 1 was applied to each correction format separately (one model to question-answer, one to fact-only etc.)

#### Research question 2

To compare the correction formats we used:

$$Model\;2:Myth\_agreement\sim correction\ast baseline\ast timepoint\;+\;(1+timepoint\vert participant)\;+\;(1+correction\ast baseline\ast timepoint\parallel myth)$$

Where correction is a fixed factor with three levels (question-answer, fact-only, fact-myth), baseline is a continuous covariate corresponding to baseline scores for each participant and myth, and timepoint is a fixed factor with two levels (timepoint 1 and timepoint 2). The * strings include all main effects and interactions for the listed factors. The model includes all main effects and interactions for fixed and random effects. Correlations between intercepts and slopes were supressed for myth random effects (identified by double pipe, ||, using lmer_alt(); see Analysis approach above).

Baseline was included as a covariate to resolve problems associated with variable degrees of belief in the myths. Myths that were not believed by participants (low baseline scores) could not be corrected (agreement scores lowered) by the intervention, and myths believed too much (high baseline scores) could not exhibit a backfire effect (agreement scores raised). Including baseline as a covariate meant that we could understand effects of the intervention at different levels of baseline belief.

To replicate the results for timepoint 1 with the secondary set of participants, we simply restricted Model 2 to timepoint 1 only:

$$Model\;2(a):Myth\_agreement\sim correction\ast baseline+(1\vert participant)+(1+correction\ast baseline\parallel myth)$$

## Results

### Research question 1: which formats are effective immediately and after a delay?

**Fig. 2:**
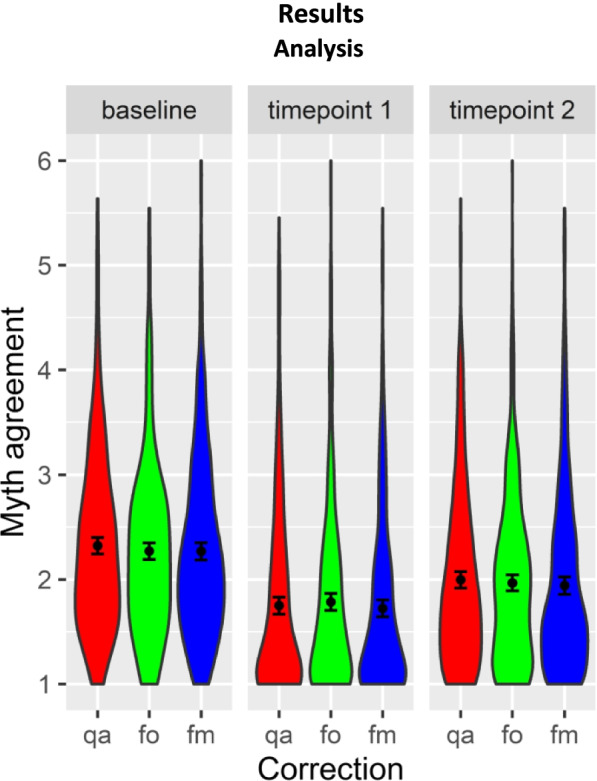
Means of myth agreement ratings (1 denotes low agreement, 6 denotes high agreement) with by-participant standard errors and violin distributions. Ratings were reduced at both timepoints 1 and 2 for all correction formats (question-answer, qa, fact-only, fo, fact-myth, fm) relative to baseline. At timepoint 2, myth agreement was higher than at timepoint 1, but stayed below baseline for all formats

Timepoint 1 and timepoint 2 myth agreement ratings were significantly lower than baseline for all correction formats (Fig. 2, see SI. Analysis Table SI.A.1 for means and Table SI.A.2 for model parameters), all β’s > 0.30, SE’s < 0.092, df’s > 11, *t*’s > 5.95, *p*’s < .001 (replication set: all β’s < 0.43, SE’s < 0.076, df’s > 11, *t*’s < − 6.95, *p’s* < .001). That is to say, each format was effective and did not backfire.

Nonetheless, ratings partially returned towards baseline at timepoint 2, as shown by significant timepoint 1 to timepoint 2 differences, all β’s > 0.18, SE’s < 0.045, df’s > 13, *t*’s > 4.6, *p*’s < .001 (although still falling short of baseline).

### Research question 2: which is the most effective correction format?

**Fig. 3:**
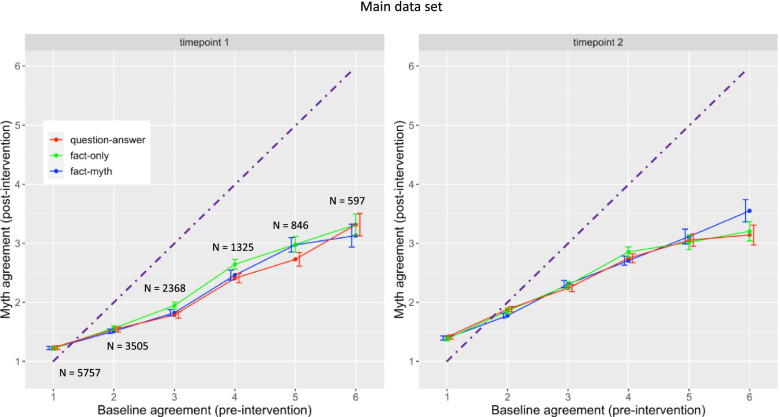
Main data set. Means of myth agreement (post-intervention) as a function of baseline agreement (pre-intervention), correction format and timepoint e.g., responses at timepoint 1 in the question-answer condition that were 2 at baseline (pre-intervention) had an average of 1.5 post-intervention. N’s indicate the number of responses in each data point e.g., there were 3505 responses that had baseline 2. No N’s are included for timepoint 2 because the same number of responses were used for timepoint 1 and timepoint 2. Dashed line shows equivalence between baseline and myth agreement (post-intervention) so that data below the line indicates correction. In both timepoints there was a strong positive correlation between baseline agreement and post-intervention agreement (post-intervention agreement was high when baseline agreement was high). Differences between correction formats were more apparent at higher levels of baseline agreement than at lower levels, hence interactions between baseline and correction format. At timepoint 1, no differences between correction formats were visible when baseline was low, but at higher levels fact-only was less effective at lowering agreement than question-answer or fact-myth (*p* = .022). At timepoint 2, again no differences were visible at low baselines, but fact-myth was less effective than question-answer when baseline was very high (*p* = .031)

**Fig. 4:**
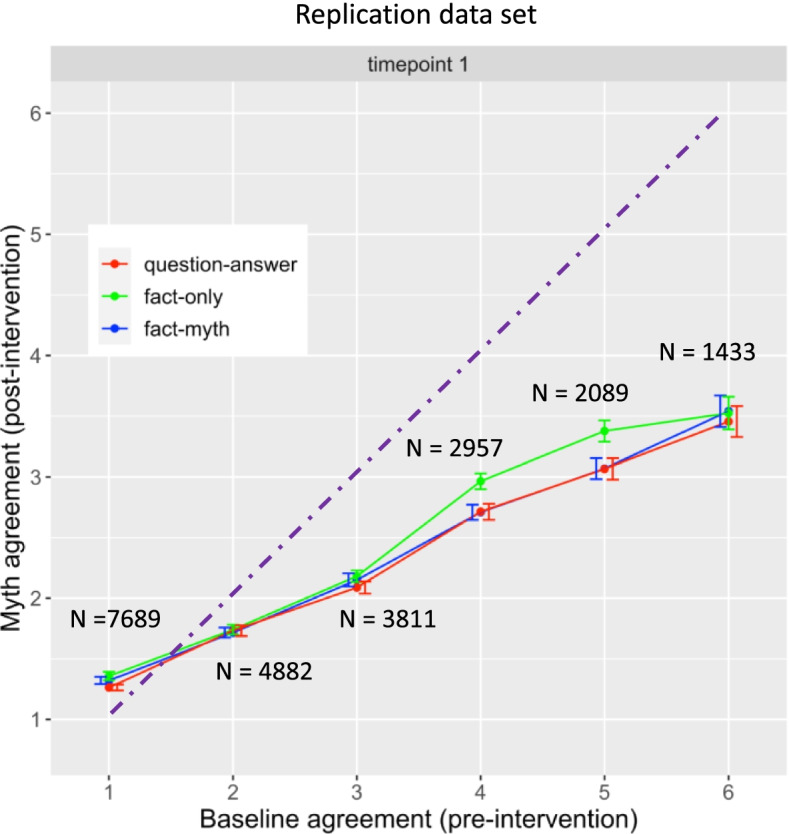
Replication data set. Means of myth agreement (post-intervention) as a function of baseline agreement (pre-intervention) and correction format. Data from replication set. N’s indicate the number of responses in each data point. Dashed line shows equivalence between baseline and myth agreement (post-intervention) so that data below the line indicates correction. Data pattern replicates main data set in that fact-only is less effective than other correction formats at higher baselines

There was no overall difference between correction formats, but there were interactions with baseline agreement and timepoint (Figs. 3 and 4). The main pattern of interest was that differences between formats became evident where the myths were more strongly believed (i.e., where baseline myth agreement was high compared to when it was low; Figs. 3 and 4). These differences are considered in detail below.

First, for question-answer vs fact-only, there was a marginal interaction with baseline and time, β = 0.032, SE = 0.018, df = 1272, *t* = 1.79, *p* = .073, such that differences were greater at higher baselines and at timepoint 1. Simple effects confirmed (and replicated) that question-answer was more effective at reducing myth agreement than fact-only for higher baselines at time point 1, β = 0.040, SE = 0.018, df = 28, *p* = .022 (replication set: β = 0.053, SE = 0.018, df = 19, *p* = .0075), but not at timepoint 2, β = 0.0075, SE = 0.019, df = 26, *t* = 0.39, *p* = 0.70.

There was also a marginal effect of question-answer vs fact-myth by baseline and time, β = − 0.020, SE = 0.010, df = 5341, *t* = − 1.93, *p* = .053, with effects smaller at timepoint 1 than timepoint 2. This was also confirmed by simple effects: there was a significant effect at timepoint 2, β = 0.040, SE = 0.018, df = 28, *p* = .031, such that question-answer was more effective than fact-myth at higher baselines compared to lower baselines. There was no significant question-answer vs fact-myth by baseline interaction at timepoint 1, β < .001, SE = 0.018, df = 35, *t* = 0.040, *p* = 0.97 (replication set: β = − 0.0028, SE = 0.013, df = 24, *p* = .84).

Finally, there was a fact-only vs fact-myth by baseline and time interaction, β = − 0.038, SE = 0.010, df = 15,990, *t* = − 3.70, *p* < .001. This was reflected as a significant simple effect at time 1 for the fact-only vs fact-myth by baseline interaction, β = − 0.051, SE = 0.017, df = 42, *t* = − 2.91, *p* = .0059 (replication set: β = − 0.061, SE = 0.016, df = 19, *t* = − 3.91, *p* < .001), such that fact-myth was more effective than fact-only at higher baselines than lower baselines. At timepoint 2 the difference between fact-only and fact-myth was no longer significant β = .025, SE = 0.015, df = 23,340, *t* = 1.63, *p* = 0.10.

#### Analysis of timepoint 1 with combined data set

**Fig. 5:**
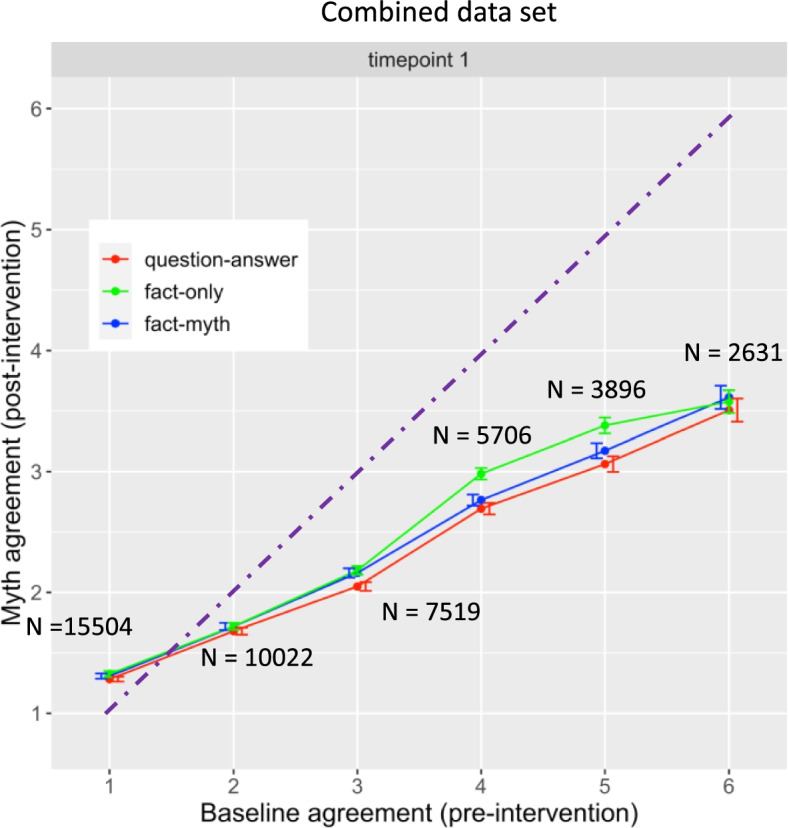
Means of myth agreement (post-intervention) as a function of baseline agreement (pre-intervention), correction format and timepoint. Data combined from complete main and replication data set. Dashed line shows equivalence between myth agreement (post-intervention) and baseline. There are interactions of correction format by baseline such that fact-only is less effective than other formats at higher baselines

The analyses above used data from the main set that included only those participants who completed both timepoints. This was necessary to allow comparison between timepoint 1 and timepoint 2. However, the consequence was a substantial loss in power when considering timepoint 1 alone (*N* = 2177 vs *N* = 1291). Furthermore, the main data set (Fig. 3) and the replication data set (Fig. 4) were analysed separately whereas as a combined analysis would have maximised power. We therefore combined the complete main data set, N = 2177, and the replication set, *N* = 2084, to yield the largest possible data set (Fig. 5).

The results replicated the individual analyses above. There were no main effects but there were interactions with baseline (Fig. 5). For question-answer vs fact-only, there was an interaction with baseline agreement, β = .039, SE = 0.012, df = 16, *t* = 3.32, *p* = 0.0044, such that question-answer was more effective at reducing myth agreement than fact-only for higher baselines. Similarly, for fact-myth vs fact-only, there was an interaction with baseline, β = .033, SE = 0.012, df = 16, *t* = 2.75, *p* = 0.015, such that fact-myth was more effective at reducing myth agreement than fact-only. There was no interaction of question-answer vs fact-myth by baseline, however, β = 0.0086, SE = 0.011, df = 16, *t* = 0.75, *p* = 0.46.

### Exploratory questions

We considered the effects of age as a non-preregistered exploratory question (analysis shown in SI.Analysis). Following Vijaykumar et al., we divided participants into an older group (> 55 years old) and a younger group (< 56 years old). Overall, older participants had lower baseline agreement for myths than younger people, consistent with Vijaykumar et al. [46], although correction was effective for all formats and no backfire effects were observed [46]. Analysis of older and younger participants separately showed that while younger participants showed the same correction format effects as those of the main analysis, older participants showed no differences.

## Discussion

This study demonstrates that simple, poster-like images, of the style used in public health campaigns, can reduce COVID-19 myth agreement both immediately post-intervention and after a delay. This efficacy applied across a UK representative sample for age and gender, across a range of myths, and was replicated in a partial study. Furthermore, it was present in older and younger people [46].

All formats proved effective at reducing myth agreement. Nonetheless, there were differences between formats where baseline (pre-intervention) myth agreement was high. Immediately post-intervention, question-answer and fact-myth were more effective correction formats than fact-only, and after a delay, question-answer was more effective than fact-myth. We therefore recommend question-answer as the preferred format for myth-busting COVID-19, all else being equal.

### No backfire effects

Misinformation researchers have sometimes observed “backfire” effects, whereby attempted correction leads to elevated belief in the myths [60–62]. While such effects have not been consistent in myth-busting research [31, 32, 35, 42], backfire was recently observed for older people when attempting to correct a COVID-19 myth about garlic [46] in a similar study to ours. We found that common COVID-19 correction formats did not cause backfire effects, even in older people.

### Correction formats

We found no main effect differences between correction formats but there were interactions with baseline agreement. These were such that differences were visible when baseline agreement was high (i.e., only when people believed the myths pre-intervention).

Immediately post-intervention, fact-myth was more effective than fact-only. This is consistent with prior studies [35, 42, 63] demonstrating that reminding participants of misinformation facilitated correction. This could be because restating the myth allows improved coactivation of the myth and the correction [24]. Another possibility is that restating the myth makes the fact more familiar relative to fact-only. Informing people that a proposition is a myth communicates that the negation of the proposition is a fact. For example, the utterance, “Some people incorrectly believe that the COVID-19 vaccine will change your DNA,” is logically equivalent to saying that the COVID-19 vaccine will not change your DNA. Thus, the advantage of fact-myth might arise because the fact is communicated more often than in fact-only.

Question-answer was also more effective than fact-only immediately post-intervention. One potential explanation is that the question-answer image motivated readers to search for a relevant myth, much like an internally motivated myth restatement. However, effects after the delay provide some evidence that this account is incorrect. Here, question-answer was more effective than fact-myth; if question-answer participants benefitted from an internal myth restatement, we should not have observed differences between the external (fact-myth) and internal (question-answer) myth restatement conditions. Note, however, that the statistical differences between question-answer and fact-myth after the delay were weak (*p* = .03) and differences were only visible at very high baseline scores (Fig. 3). Further evidence is required to confirm the advantages of question-answer at longer intervals.

Another possibility is that the question-answer advantage arose from facilitated retrieval and/or encoding. Retrieval might have been facilitated in a similar way to the testing effect seen in educational settings [64]. In educational research, it is well known that self-testing (questioning oneself about the to-be-learnt material) produces better long-term recall than repeated reading of the to-be-learnt material, one explanation being that testing enhances learning by producing elaboration of existing memory traces and their cue-target relationships [65]. However, it is unclear whether immediately providing the answer, as in the question-answer format used here, is equivalent to providing the answer after a delay, as is typical in educational research.

The discourse structure was different in question-answer than fact-myth or fact-only. This could have contributed to encoding differences. First, question-answer was pragmatically more felicitous than fact-only. Fact-only lacked an obvious “question-under-discussion” [66], a reason why the fact was presented, and so participants were obliged to expend effort in search of one. Second, question-answer provided a clear statement about the veracity of the queried fact. The answer (“yes” or “no”) told participants whether the statement was true and might have acted as a memory “tag” [18, 67]. In other conditions, the veracity of the fact had to be inferred from the experimental context.

In summary, question-answer and fact-myth conferred advantages relative to fact-only immediately post-intervention. After the delay, there was some evidence that question-answer was more effective than fact-only. These findings lead us to recommend question-answer as the preferred format for COVID-19 myth correction campaigns (in contrast to the format used by some current campaigns, e.g., WHO [30], which use fact-only formats). However, it is important to emphasise that the effects of correction format were small compared to the effects of correction more generally (compare differences across correction formats in Fig. 2 with differences between baseline and post intervention), especially after the delay. It is thus better to include correction in any format than to avoid doing so for fear of causing harm with an ineffective myth-busting campaign (see [68] for similar point).

### Limitations and future studies

Our study comes with a number of caveats and opportunities for further research. The first relates to the myths we tested. The level of belief in our myths was low overall, around 40% at baseline, which meant that there was only limited room for correction (although much room for backfire effects). The consequence was that the power of the study was reduced relative to a study with more strongly believed myths (e.g., [31]) and this may have contributed to our failure to find differences between some conditions. Nonetheless, the loss in power was accompanied by a gain in validity. The materials we used were genuine COVID-19 myths, recruited from fact-checker websites, rather than the everyday narratives used in continued influence paradigms [42]. The results of this study are therefore more likely to generalise to COVID-19 myth-busting campaigns than if we had used non-COVID materials.

By limiting our materials to myths found in current COVID-19 health information, we not only limited the pre-existing myth belief, we also limited the range of myths. The myths we tested could all be considered *rumours* [69], in that they were factually verifiable and designed not to inflame political beliefs. Our conclusions are thus limited to these forms of misinformation. Other types of misinformation, such as conspiracy theories, tend to be much more difficult to correct [70] and may respond differently to the correction formats we tested.

The second limitation relates to the degree of engagement with the materials. Our findings are the result of participants reading each correction image when told to do so, independently of whether they found the topic or format engaging. In real health campaigns, people will only process material they are drawn to engage with, and the danger is that engagement and memory will dissociate so it remains possible that, for example, question-answer produces the most effective memory correction, but fact-myth is more engaging.

Relatedly, we did not test the effects of partial engagement. Many readers outside of an experiment will only shallowly process posters or social media content, perhaps just reading the title [71] or initial sentences, or their attention might be divided between reading the correction and other tasks, impairing memory [72] and even the processing of corrections specifically [73]. Correction under these conditions may be weaker than effects reported here [33] and may differ according to correction format. For example, were people to read the question of a question-answer format poster without reading the answer (“Does the COVID-19 vaccine change your DNA?”), the myth may become more familiar than if the question had not been read at all. This would be more likely when the answer was separated from the question by large chunks of intervening material.

Finally, there were subtle differences between the formats we tested and the materials used in health campaigns. These differences may influence the extent to which our findings generalise. The first is that where we included myths, we did so *after* stating the fact, in line with current guidance. However, health campaigns, fact checkers, and previous studies have often presented the myth first and then the fact [7, 62]. From a partial engagement perspective putting the fact first reduces the probability that the myth is read without the correcting fact, although a recent pre-print [68] found myth-fact to be more effective than fact-myth after a delayed retention. Second, we did not use the word “myth”, as in the traditional myth-busting format (“Myth: The COVID-19 vaccine changes your DNA”). Instead, we used synonymous text strings (“Some people wrongly believe that…”) that fitted better with the structure of the materials and is widely employed in campaigns (e.g., [74]). It is possible that using the more concise, lexical form would be easier for people to process and so lead to greater correction.

## Conclusion

Our results imply that COVID-19 myths can be effectively corrected using materials and formats typical of health campaigns [25, 26]. This applies across subgroups for whom backfire effects have previously been observed [46]. Health campaigns can also use our results to select the optimum correction formats. While myth-busting in any of the three formats we tested was effective, question-answer format and fact-myth were more effective than fact-only, and there was some evidence that question-answer was more effective than fact-myth in the longer term. Further research needs to widen the range of myths tested from the verifiable rumours considered here to conspiracy theories [69], and to consider how different formats behave under partial engagement conditions.

## Supplementary Information

**Additional file 1.** Supplementary Information: Methods.

**Additional file 2.** Supplementary Information: Analysis.

## Data Availability

The datasets generated and analysed during the current study are available in the Open Science Framework repository, https://osf.io/huz4q/.

## Abbreviations

WHO: World Health Organization
LME: Linear mixed effect
