# Supplementary material for: COVID-19 myth-busting: an experimental study
Source: BMC Public Health. 2022 Jan 19;22:131. doi: 10.1186/s12889-021-12464-3 (PMC8767039; doi:10.1186/s12889-021-12464-3)
Supplement: Supplementary file 1 — Additional file 1. Supplementary Information: Methods. [file 12889_2021_12464_MOESM1_ESM.docx]

**Supplementary Information: Methods**

|  | Fact-myth | Fact-only | Question-answer | Explanation | Agreement Question |
| --- | --- | --- | --- | --- | --- |
| 1 | A COVID-19 test will not show a positive result if you only have a cold or flu.  A common COVID-19 myth is that seasonal colds and flu are wrongly being counted as COVID-19 cases. | A COVID-19 test will not show a positive result if you only have a cold or flu. | Will a COVID-19 test show a positive result if you only have a cold or flu?  No. | The COVID-19 swab (PCR, antigen) test has been specifically developed to detect the presence of COVID-19 only. It has a proven accuracy rate of 99.91%. A cold or the flu won't cause positive tests for coronavirus, even though symptoms can be similar. | If I have the flu, I will be included in the number of COVID-19 cases |
| 2 | If you have any COVID-19 symptoms, however mild, you should take a test and stay at home.  A common myth is that if your symptoms are mild, you can self-isolate and don't have to take a test. | If you have any COVID-19 symptoms, however mild, you should take a test and stay at home. | If you only have mild COVID-19 symptoms, do you still have to take a test and stay at home?  Yes. | Even if your symptoms are mild, you can still pass COVID-19 on to others. Symptoms include a high temperature, a new continuous cough and a loss or change to your sense of smell or taste. | If my symptoms are mild, I don’t need to take a test and can just stay at home. |
| 3 | Lockdowns can slow COVID-19 transmission.  Some people wrongly believe that lockdowns don’t work. | Lockdowns can slow COVID-19 transmission. | Do lockdowns slow COVID-19 transmission?  Yes. | Lockdowns limit contact between people which is important to slow the spread of the disease. The first lockdown, which resulted in a very large reduction in mobility, was followed by a reduction in COVID-19 cases. | I do not believe that lockdowns are effective. |
| 4 | Thermal scanners measure a person’s temperature and not whether they have COVID-19.  A common myth is that thermal scanners can detect people who have COVID-19 but aren’t showing symptoms. | Thermal scanners measure a person’s temperature and not whether they have COVID-19. | Can thermal scanners detect people who have COVID-19 but aren’t showing symptoms?  No. | Thermal scanners detect people whose temperatures are higher than normal.  If you think your temperature is higher than normal, you should book an NHS swab test. | Thermal scanners can detect whether I have COVID-19 even if I am not showing symptoms |
| 5 | COVID-19 is more deadly than the flu.  Some people wrongly believe that COVID-19 is just like the flu and causes no more deaths. | COVID-19 is more deadly than the flu. | Is COVID-19 more deadly than the flu?  Yes. | Despite the numerous restrictions on public movement (which aren’t enforced during flu seasons), COVID-19 deaths have been higher than flu deaths during recent bad flu seasons. | I do not believe that COVID-19 causes more deaths than the flu. |
| 6 | The COVID-19 vaccination is not mandatory.  Some people wrongly believe that it is. | The COVID-19 vaccination is not mandatory. | Is the COVID-19 vaccination mandatory?  No. | There are no mandatory vaccines in the UK. You can choose whether to have them. Nevertheless, vaccines are an important way to keep ourselves, friends and family safe. | It is mandatory for me to have my COVID-19 vaccination |
| 7 | Avoiding a COVID-19 test to keep official numbers down will not stop lockdowns.  Some people wrongly believe that the lockdowns are due to healthy people getting tested. | Avoiding a COVID-19 test to keep official numbers down will not stop lockdowns. | Will avoiding a COVID-19 test keep official numbers down and stop lockdowns?  No. | You should get a test if you have symptoms. The number of tests and positive cases are used to measure the scale of an outbreak in a certain area. A lack of testing might lead to an extended pandemic. | If I avoid taking a COVID-19 test I can help prevent lockdowns from happening |
| 8 | Ultra-violet lamps should not be used to disinfect hands.  Some people wrongly believe that they can. | Ultra-violet lamps should not be used to disinfect hands. | Should ultra-violet lamps be used to disinfect hands?  No. | UV radiation should not be used to disinfect hands as UV radiation can cause skin irritation and damage your eyes. You should use soap and water (or hand sanitiser gel if soap and water are not available). | It is safe for me to disinfect my hands using an ultra-violet lamp |
| 9 | Face masks reduce transmission of COVID-19.  Some people wrongly believe that face masks do not reduce the transmission of infection. | Face masks reduce transmission of COVID-19. | Do face masks reduce the transmission of COVID-19?  Yes. | Face masks reduce the spread of respiratory droplets and small aerosols that carry COVID-19 from an infected person into the air. They also provide some protection for the wearer against droplets. | I believe that face masks will not reduce the spread of my respiratory droplets and transmission of COVID-19 |
| 10 | Wearing a face mask does not lead to increased levels of carbon dioxide in the blood.  Some people wrongly believe that wearing a mask makes you breathe in too much carbon dioxide and so makes you ill. | Wearing a face mask does not lead to increased levels of carbon dioxide in the blood. | Does wearing a face mask lead to increased levels of carbon dioxide in the blood?  No. | Some people may find face masks uncomfortable, but they are not harmful if you are fit and well. | I believe wearing a face mask will lead to higher levels of carbon dioxide in my blood |
| 11 | After you have had the vaccine, you cannot immediately return to the life you had before  COVID-19.  Some people wrongly believe that as soon as they have had the vaccine, they can go back to their normal life. | After you have had the vaccine, you cannot immediately return to the life you had before  COVID-19. | After I have had the vaccine, can I immediately return to the life I had before COVID-19?  No. | Even when COVID-19 vaccines are rolled-out, it will take a while to reach everyone. You will still be asked to follow other public health measures such as socially distancing, wearing a mask and limiting social mixing. | I can return to normal life immediately if I have had my COVID-19 vaccine. |

Table SI.M.1 Study materials. Participants saw text from one of fact-only, fact-myth, question-answer columns, and also the explanation statement.

| Fact agreement questions | I can protect others by regularly washing my hands with soap and water. |
| --- | --- |
|  | To help stop the spread of COVID-19, I can wash my hands with hand sanitiser gel. |
|  | Symptoms of the coronavirus include a new continuous cough and/or a high temperature. |
|  | I should not touch my eyes, nose or mouth if my hands are not clean. |
| Catch questions | There are seven days in the week. |
|  | The first letter of the alphabet is ‘T’. |

Table SI.M.2. Fact and catch questions.

| Demographic information | Please enter your age  Please enter your gender  Please enter the highest level of education you have completed. e.g. if you have completed your A-levels and are currently doing a degree, you would select "A-levels" |
| --- | --- |
| COVID-19 experiences | Are you a healthcare worker?  According to the NHS advice, are you in a higher risk group for the coronavirus?  (This group includes older people, people with health conditions and pregnant women)  Have you had symptoms of the coronavirus? |
| Vaccine concern | How concerned are you about serious side effects from vaccines? |
| Vaccine intentions | How likely is it that you will get a COVID-19 vaccine?  How likely is it that you will get a flu vaccine for the seasonal flu during the upcoming flu season (autumn 2021–spring2022)? |

Table SI.M.3. Demographic, COVID-19 experiences and vaccine intentions questions.
