# Supplementary material for: COVID-19 myth-busting: an experimental study
Source: BMC Public Health. 2022 Jan 19;22:131. doi: 10.1186/s12889-021-12464-3 (PMC8767039; doi:10.1186/s12889-021-12464-3)
Supplement: Supplementary file 2 — Additional file 2. Supplementary Information: Analysis. [file 12889_2021_12464_MOESM2_ESM.docx]

**Supplementary Information: Analysis**

**Tables**

|  |  | **Baseline** | | **Timepoint 1** | | **Timepoint 2** | |
| --- | --- | --- | --- | --- | --- | --- | --- |
| **Data Set** | **Condition** | **Mean** | **SD** | **Mean** | **SD** | **Mean** | **SD** |
| **Main** | Question-answer | 2.320 | 1.439 | 1.751 | 1.269 | 1.996 | 1.327 |
|  | Fact-only | 2.269 | 1.446 | 1.785 | 1.305 | 1.970 | 1.357 |
|  | Fact-myth | 2.268 | 1.424 | 1.723 | 1.222 | 1.941 | 1.318 |
| **Replication** | Question-answer | 2.623 | 1.567 | 2.090 | 1.510 | n/a | |
|  | Fact-only | 2.583 | 1.562 | 2.152 | 1.526 |  |  |
|  | Fact-myth | 2.637 | 1.586 | 2.119 | 1.539 |  |  |

Table SI.A.1. Means and standard deviations (SD) from the Main and Replication data sets for the three conditions (question-answer, fact-only, fact-myth) and timepoints (baseline, timepoint 1, timepoint 2)

| **Correction format** | **Data set** | **Comparison** | **β** | **SE** | **df** | ***t*** | ***p*** |
| --- | --- | --- | --- | --- | --- | --- | --- |
| Question-answer | Main | Timepoint 1 vs baseline | -0.57 | 0.084 | 10.69 | -6.83 | < .001 |
|  |  | Timepoint 2 vs baseline | -0.32 | 0.055 | 11.98 | -5.95 | < .001 |
|  |  | Timepoint 1 vs Timepoint 2 | 0.25 | 0.045 | 13.00 | 5.47 | < .001 |
|  | Replication | Timepoint 1 vs baseline | -0.53 | 0.076 | 10.63 | -6.95 | < .001 |
| Fact-only | Main | Timepoint 1 | -0.48 | 0.059 | 10.90 | -8.26 | < .001 |
|  |  | Timepoint 2 | -0.30 | 0.046 | 16.13 | -6.62 | < .001 |
|  |  | Timepoint 1 vs Timepoint 2 | 0.18 | 0.040 | 14.99 | 4.59 | < .001 |
|  | Replication | Timepoint 1 vs baseline | -0.43 | 0.047 | 10.92 | -9.13 | < .001 |
| Fact-myth | Main | Timepoint 1 vs baseline | -0.55 | 0.061 | 11.11 | -8.93 | < .001 |
|  |  | Timepoint 2 vs baseline | -0.33 | 0.040 | 14.77 | -8.19 | < .001 |
|  |  | Timepoint 1 vs Timepoint 2 | 0.22 | 0.038 | 14.64 | 5.67 | < .001 |
|  | Replication | Timepoint 1 vs baseline | -0.52 | 0.066 | 11.00 | -7.88 | < .001 |

Table SI.A.2. Table showing final models

**Effects of age**

**Rationale for analysis.** Myth belief has been reported to differ between older and younger participants, and backfire effects may be more likely for older participants, but a consistent pattern to guide health campaigns remains elusive. In a recent study Vijaykumar et al. (1) found younger participants (18-54) had higher belief for COVID-19 misinformation, which appears to reverse the previous narrative that older participants are more vulnerable to misinformation (2). For example, older participants were previously found to express more confidence in false memories (3), and share more fake news or links to political misinformation (4,5). It is possible this reflects a divergence between health myths and other kinds of misinformation, if older participants have accumulated more health or science knowledge to rely upon. However, there has also been a backfire effect reported for COVID-19 myth-busting in older adults, at least in the UK, where misinformation belief actually increased after viewing the corrective materials (1). Likewise, one of the earliest papers on the familiarity backfire effect (6) found that one week after reading material correcting influenza myth correction, older participants had greater belief in the myths than prior to intervention but younger participants did not, although Swire et al., (7), failed to find backfire effects in older participants.

**Analysis and Results.** To compare older and younger participants, we created a new factor, age_group, which divided participants into younger (<56 years old, *n* = 813) or older (>55, *n* = 481) participants (following the distinction made by Vijaykumar et al. (1)). We then combined this factor with Model 2:

Model 3: Myth_agreement ~ correction*baseline*timepoint*age_group + (1+timepoint|participant) + (1+correction*baseline*timepoint*age_group||myth)

where age_group is a fixed effect with appropriate random effects. Correlations were suppressed for myth random effects. Analysis was on the main set only, not the replication set.

Fig SI.A.1. Myth agreement ratings for (A) younger participants and (B) older participants. For both groups, the interventions (question-answer, qa, fact-only, fo, fact-myth, fm) successfully lowered agreement at both timepoints. Younger participants had more varied and higher average ratings than older participants but a shallower increase from timepoint 1 to timepoint 2.

Younger

Fig SI.A.2. Means of myth agreement (post-intervention) as a function of baseline agreement (pre-intervention), correction format, and time, for younger participants. Error bars are participant standard errors by correction format and baseline. N’s indicate the number of responses that constitute each data point. Dashed line shows equivalence between baseline and myth agreement (post-intervention) so that data below the line indicates effective correction. There were interactions of correction format and baseline agreement at both timepoints. At timepoint 1, fact-only was less effective than question-answer and fact-myth at higher baselines. At timepoint 2, fact-myth was less effective than question-answer.

Older

Fig SI.A.3. Means of myth agreement (post-intervention) as a function of baseline agreement (pre-intervention), correction format, and time, for older participants. Error bars are participant standard errors by correction format and baseline. N’s indicate the number of responses that constitute each data point. Dashed line shows equivalence between baseline and myth agreement (post-intervention) so that data below the line indicates effective correction. There were interactions of correction format and baseline agreement at both timepoints. At timepoint 1, fact-only was less effective than question-answer and fact-myth at higher baselines. At timepoint 2, fact-myth was less effective than question-answer.

There were stark differences in agreements ratings across age (Fig SI.A.1, Fig SI.A.2, Fig SI.A.3). Younger participants showed higher agreement overall than older participants, β = -0.12, SE = 0.018, df = 131, *t* = -6.78, *p* < .001, and there was an interaction of age by time, β = -0.029, SE= 0.0065, df = 12.17, *t* = -4.42, *p* < .001, with the increase from timepoint 1 to timepoint 2 being greater in older participants (Fig SI.A.2 & Fig SI.A.1). Because there were significant three and four-way interactions involving age, correction format, baseline and time (age by question-answer *vs* fact-only by baseline, β = -0.11, SE = 0.028, df = 41, *t* = -3.96, *p* < .001; age by question-answer *vs* fact-myth by baseline β = 0.085, SE = 0.031, df = 22, *t* = 2.70, *p* = 0.013; age by fact-only *vs* fact-myth by baseline by time, β = -0.044, SE = 0.022, df = 19490, *t* = -2.01, *p* = 0.045), we consider the effects of correction format on younger and older participants separately (convergence issues prevented us from estimating the simple effects of age from Model 2).

*Younger participants*

There was a significant question-answer *vs* fact-only by baseline by time interaction, β = -0.063, se = 0.026, df = 9128, *p* = 0.014. Simple effects showed that at timepoint 1, there was a significant question-answer *vs* fact-only by baseline interaction (Fig SI.A.2), β = 0.097, SE = 0.024, df = 29, *t* = 4.01, *p* < .001, such that question-answer was more effective than fact-only at higher baselines, but at timepoint 2 there was not, β = 0.032, SE = 0.023, df = 25, *t* = 1.39, *p* = 0.18.

There was a marginal question-answer *vs* fact-myth by baseline by time interaction, β = 0.044, SE = 0.026, df = 10010, *p* = 0.09, with effects larger at timepoint 2. At timepoint 1, there was no significant question-answer *vs* fact-myth by baseline interaction, β = 0.024, SE = 0.022, df = 45, *t* = 1.13, *p* = 0.26, but there was at timepoint 2, β = -0.074, SE = 0.021, df = 56, *t* = -3.49, *p* < .001, such that question-answer was more effective at reducing myth agreement than fact-myth at higher baselines.

Finally, there was a significant fact-only *vs* fact-myth by baseline by time interaction, β = 0.11, SE = 0.027, df = 10320, *t* = 4.01, *p* < .001. At timepoint 1, there was a fact-only *vs* fact-myth by baseline interaction β = -.074, SE = 0.021, df = 56, *t* = -3.49, *p* < .001, with fact-only less effective than fact-myth at higher baseline, but at timepoint 2, there was no difference, β = -0.036, SE = 0.023, df = 29, *t* = -1.59, *p* = 0.12.

*Older participants*

For older participants, importantly, there were no backfire effects (Fig SI.A.3). There were also no interactions with format. There was a significant main effect of time, β = 0.24, SE = 0.042, df = 12, *t* = 5.87, *p* < .001, such that agreement increased from timepoint 1 to timepoint 2.

**Discussion**

There were stark differences in myth belief between age groups. Older people had substantially lower belief in myths than younger people, consistent with Vijaykumar (1) and (8), both at baseline and after correction. The convergence across studies suggests that these findings are not due to idiosyncratic choices of myths but represents a general pattern in the belief of misinformation. One explanation is that older people are able to engage their more extensive general knowledge to discount new information (9), as argued by Vijaykumar (1), but it is also possible that they are exposed to a smaller range of myths, with less frequent repetition, than younger people, due to lower engagement with social media.

Although we replicated the age differences in myth belief observed by Vijakumar, we observed clear positive correction effects in older people (SI.A.1) rather than backfire effects. Differences could have arisen because of the materials used. Vijaykumar et al. used only one myth (the curative properties of garlic), whereas we used eleven (of which none concerned garlic), and it is possible that only certain myths yield backfire effects. Vijaykumar et al. also reinforced the myth prior to correction, which we did not. There may therefore be an interaction between age, reinforcement and correction that was present in Vijaykumar but not in our study.

Myth agreement in older participants was corrected at similar proportions to younger participants immediately post-intervention. However, beliefs returned towards baseline at a faster rate than for younger participants, perhaps because correcting beliefs requires strategic memory processes, and these are less efficient in older people (7,10).

Correction format effects were present in younger participants - more clearly than in the main analysis - but not in older participants. Furthermore, there were interactions with age that indicate that this was not only due to lower baseline beliefs in the older group but to the participants themselves. One explanation is that there were floor effects in the older group but not the younger group: older participants were at minimum agreement levels at timepoint 1, whereas younger participants were not. However, it is clear that by timepoint 2, older participants were not at floor level, and at timepoint 2 no effects of correction format were visible in older participants but they were in younger participants. It is therefore possible that the differing cognitive abilities of older participants are responsible for the insensitivity to correction format.

**References**

1. Vijaykumar S, Jin Y, Rogerson D, Lu X, Sharma S, Maughan A, et al. How shades of truth and age affect responses to COVID-19 (Mis)information: randomized survey experiment among WhatsApp users in UK and Brazil. Humanit Soc Sci Commun. 2021 Mar;8(1):1–12.
2. Wylie LE, Patihis L, McCuller LL. Misinformation Effect in Older Versus Younger Adults A Meta-Analysis and Review. Psychology Press; 2014. 38–66 p.
3. Jacoby LL, Rhodes MG. False remembering in the aged. Current Directions in Psychological Science. 2006 Apr;15(2):49-53.
4. Grinberg N, Joseph K, Friedland L, Swire-Thompson B, Lazer D. Fake news on Twitter during the 2016 US presidential election. Science. 2019 Jan 25;363(6425):374-8.
5. Guess A, Nagler J, Tucker J. Less than you think : Prevalence and predictors of fake news dissemination on Facebook. 2019;(January):1–9
6. Skurnik I, Yoon C, Park DC, Schwarz N. How Warnings about False Claims Become Recommendations. J Consum Res. 2005;31(4):713–24.
7. Swire B, Ecker UKH, Lewandowsky S. The role of familiarity in correcting inaccurate information. J Exp Psychol Learn Mem Cogn. 2017;43(12):1948–61.
8. Baum MA, Ognyanova K, Chwe H, Quintana A, Perlis R, Lazer D, Druckman J, Santillana M, Lin J, Volpe J, Simonson M. The State of the Nation: A 50-State Covid-19 Survey Report# 14: Misinformation and Vaccine Acceptance. Homeland Security Digital Library. 2020.
9. Umanath S, Marsh EJ. Understanding how prior knowledge influences memory in older adults. Perspectives on Psychological Science. 2014 Jul;9(4):408-26.
10. Prull MW, Dawes LL, Martin III AM, Rosenberg HF, Light LL. Recollection and familiarity in recognition memory: adult age differences and neuropsychological test correlates. Psychology and aging. 2006 Mar;21(1):107.
